# The Role Transition of Dietary Species Richness in Modulating the Gut Microbial Assembly and Postweaning Performance of a Generalist Herbivore

**DOI:** 10.1128/mSystems.00979-21

**Published:** 2021-11-02

**Authors:** Guoliang Li, Chao Shi, Yiran Song, Haiyan Chu, Zhibin Zhang

**Affiliations:** a State Key Laboratory of Integrated Management of Pest Insects and Rodents, Institute of Zoology, Chinese Academy of Sciences, Beijing, China; b CAS Center for Excellence in Biotic Interactions, University of Chinese Academy of Sciences, Beijing, China; c College of Biological Resources and Environmental Science, Jishou University, Jishou, China; d State Key Laboratory of Soil and Sustainable Agriculture, Institute of Soil Science, Chinese Academy of Sciences, Nanjing, China; KU Leuven

**Keywords:** community assembly, diet diversity, null model, gut microbiota, global environment change

## Abstract

When facing a food shortage, generalist herbivores can respond by expanding their dietary species richness (DSR) to maximize energy collection, regardless of whether forages are preferred or not. Higher DSR usually indicates higher nutrient adequacy and better health. However, the high-DSR diet containing a large proportion of preferred species or a large proportion of less-preferred species means different things to an animal. It is still unknown how different shift patterns in DSR would affect distinctly the performance of animals via altering gut microbiota. We examined the gut microbial composition, diversity, community assembly processes, and performance of a generalist herbivore, *Lasiopodomys brandtii*, in a feeding experiment with increased levels of simulated DSR shifting from preferred plant species to less preferred ones. We found the survival rate and body growth of Brandt’s voles showed a dome-shaped association with DSR: species performance increased initially with the increase of preferred plant species but declined with the increase of less-preferred food items. Several microbial taxa and functions closely related to the metabolism of amino acids and short-chain fatty acids also showed a dome-shaped association with DSR, which is consistent with the observation of performance change. However, the alpha diversities of gut microbiota increased linearly with DSR. The null model and phylogenetic analysis suggested that stochastic processes dominate at low DSR diets, whereas deterministic processes prevail at high DSR diets. These results suggest that the role of DSR in regulating animal performance by gut microbiota depends on the number of preferred forage items.

**IMPORTANCE** The plant species diversity varies greatly under the influence of both climate change and human disturbance, which may negatively affect the productivity as well as the variability of organisms (e.g., small herbivores) at the next trophic level. It is still unknown how gut microbiota of small herbivores respond to such changes in dietary species richness. Our manipulative food experiment revealed that dietary species richness can affect the composition, functions, and community assembly of gut microbiota of Brandt’s vole in a nonlinear way. Given the fast-growing interest in therapeutic diets to treat dysbiosis and to improve health conditions, our study highlights the need to consider not just the variety of consumed food but also the principles of rational nutrition.

## INTRODUCTION

The impacts of climate change and human disturbance on plant diversity in grasslands have been subjected to growing concern (1). For example, some studies indicated a positive effect of climate warming and abundant precipitation on local plant diversity in high-altitude habitats or semi-arid grassland by favoring specific species (2–4). Human disturbance, such as moderate livestock grazing, tends to facilitate plant diversity in high-productivity areas by suppressing competitively dominant grassland plants (5, 6). Such changes in plant diversity can exert cascading effects through food webs, directly affecting higher trophic levels (e.g., small herbivores) through altered dietary species richness (DSR). As a measure of food biodiversity, DSR not only plays a fundamental role in the quality of dietary nutrition but also affects the performance and abundance of generalist herbivores (7). Higher DSR can reduce the energy cost of locating food resources and provide a balanced nutrient supply for consumers, as well as act as a buffer against stochastic loss of food species under harsh natural conditions (8). Accordingly, a long-standing hypothesis in ecology holds that lower DSR can cause serious population decline and shrinkage of wildlife (9), while greater DSR is associated with a lower risk of consumer population collapse (10–12). Amid ongoing global changes, however, a preferable level of high DSR has often been accompanied by a parallel increase in negative effects on consumers. For example, successive livestock overgrazing can significantly cause a diminution of preferred or palatable diet components for small herbivores (13, 14). The scarcity of preferred food items may lead to trophic mismatch and eventually cause negative consequences for the performance of small herbivores, which could offset the benefits of diet mixing. The assessment of the role of changed DSR in wild herbivore performance by distinguishing between the preferred and less-preferred food species is thereby needed to accurately predict plant-herbivore interactions, population range shift, and associated ecosystem dynamics under a scenario of global change.

Food preference by herbivores often depends on the contents of nutrient and secondary metabolites (15). The less-preferred plant species by herbivores usually contain high concentration of secondary metabolites that can severely hinder nutrient absorption for consumers. However, some symbiotic microbes inhabiting the gut lumen, such as Escherichia coli, Bacillus subtilis and Enterococcus faecalis can degrade secondary metabolites (e.g., hydrolyzable tannins) and help herbivores to consume tannin-rich diets (16). As a result, tannin concentrations of diets would significantly shape the composition of gut microbiota of consumers. In addition, different nutrient patterns are also associated with distinct combinations of gut microbiota. For example, high-fat diet can increase the relative abundance of *Rikenellaceae*, while it decreases the relative abundance of *Ruminococcaceae* in mice (17). Diet is considered a dominant factor causing variation in the microbiota composition (4, 18). Unfortunately, traditional approaches to diet-microbiota studies have usually focused on the effects of single nutrients, namely, those of energy (19), fat (17), or sugars (20). In contrast, rather than foraging for specific single nutrients, most wild herbivores, particularly in the face of environmental changes, consume a wide spectrum of food species that varies largely in toxins and nutrients. If food items are assumed to act independently, each of them providing distinct nutrients or sustaining unique microbial colonists is expected to have a positive influence on DSR and the resulting gut microbial diversity. To the best of our knowledge, only a handful of studies, such as those of Li et al. (21) and Bolnick et al. (22), have tested this positive relationship for two freshwater fish species (threespine stickleback [Gasterosteus aculeatus] and Eurasian perch [Perca fluviatilis]) and the wild plateau pika (Ochotona curzoniae), while the additive effect of food items on gut microbial diversity has not yet been sufficiently validated (21, 22). As such, further knowledge is needed to better understand the importance of DSR for gut microbiota diversity and functioning across a wider range of species.

The understanding of fundamental ecological processes controlling the community assembly is a key topic in microbial ecology (23). It is widely acknowledged that community assembly is mediated simultaneously by deterministic and stochastic processes (24–26). The deterministic processes mainly include ecological selection imposed by both abiotic (i.e., environmental filtering) and biotic factors (i.e., interspecific interaction) (27). In contrast, stochastic processes comprise probabilistic dispersal, unpredictable disturbance, ecological drift, and stochastic extinction events (28). In recent years, exploring the variation in the relative importance of stochastic and deterministic processes across different environments has been a subject of great interest in community ecology. For example, the balance between stochastic and deterministic elements behind the assembly of bacteria in numerous habitats has been reported to be mediated by various factors, such as soil pH (27), elevation (29), development stage (30), and species richness (31). Dietary species richness and species identity are associated with micronutrient adequacy and varied toxin intake. Interaction between nutrient concentrations and dietary toxin concentrations may have a strong downstream effect on the gut community assembly and the performance of small herbivores. Thus, in the context of global environmental change, the information obtained by exploring the variation in gut microbiota assembly along DSR gradients has significant implications for the understanding of spatiotemporal population dynamics and range shift of small herbivore species.

The Brandt’s vole is a typical herbivorous rodent species inhabiting semi-arid steppes, and oscillations in their population dynamics can have cascading effects on species interactions, food web structure and ecosystem functioning (32). Due to present climate warming and livestock grazing disturbance issues, the diet of Brandt’s vole, which used to contain a wide variety of preferred or palatable plants (e.g., Leymus chinensis and Stipa krylovii) has gradually shifted to one consisting mostly of nonpreferred plant species (e.g., Cleistogenes squarrosa and Chenopodium aristatum), even though the number of dietary species have increased. Our previous relevant studies focused on the relationship between the gut microbiome of Brandt’s vole and livestock grazing or social distress (33, 34). It is still unclear, however, whether the percentage of less-preferred food plants in the vole’s diet mediates the effect of DSR on species performance through the alteration of gut microbiome. Therefore, in this study, postweaning Brandt’s voles were kept on a diet along a gradient of DSR, and the performance features (including survival rate, body growth and immune response) of voles were subsequently measured. The gut microbial diversity and functions were profiled by 16S amplicon sequencing and shotgun metagenomic sequencing, respectively, and the small metabolites (including amino acids and short chain fatty acids) in fecal samples were quantified by quantitative targeted metabolomics to validate the changes in metabolic functions of microbiome along different DSR treatments. We hypothesize that the diets with increasing DSR, accomplished by the gradual addition of plant species to diet in descending order of preference, affects the composition, community assembly and functions of gut microbiota, thereby having a dome-shaped effect on performance of voles.

## RESULTS

### Nutritional shift in diets with different plant species richness.

**FIG 1:**
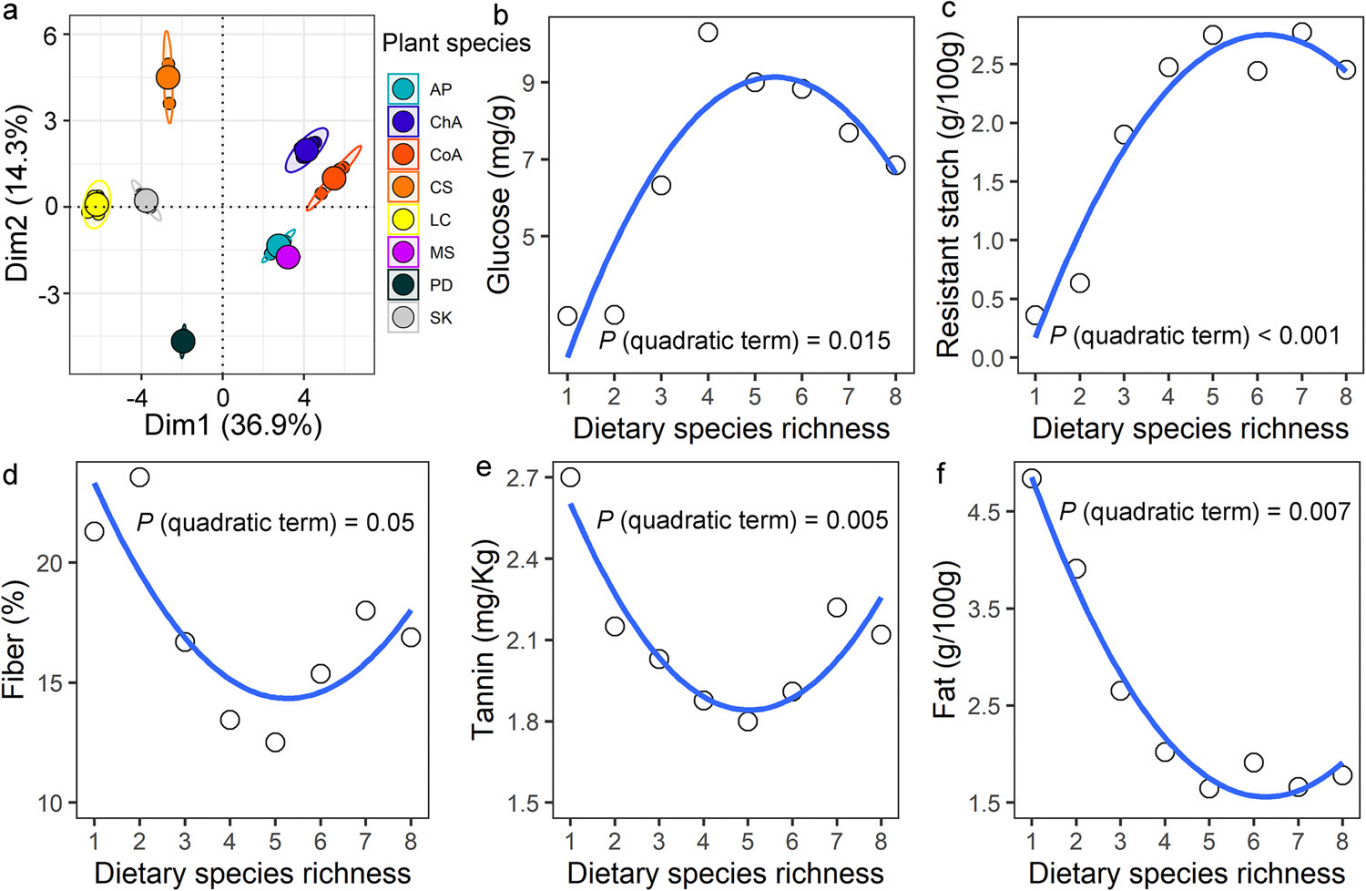
Nutritional analysis of diets with different plant species richness. (a) The difference in nutritional composition of eight plant species (*Allium polyrhizum*, AP; *Chenopodium aristatum*, ChA; *Convolvulus ammannii*, CoA; *Cleistogenes squarrosa*, CS; *Leymus chinensis*, LC; *Medicago sativa*, MS; *Phlomis dentosa*, PD; *Stipa krylovii*, SK). (b–f) The nonlinear relationship between the contents of nutritional indexes (i.e., glucose, resistant starch, fiber, tannin, and fat, respectively) and dietary species richness.

The permutational multivariate analysis of variance (PERMANOVA) indicated great differences in the nutritional composition among the 8 plant species used in our study (Fig. 1a; *F*_7,48_ = 968.8, *P < *0.001). Feeds with different plant species richness corresponded to diverse patterns of nutrient and antinutritive compound intake for voles (Fig. 1). More specifically, glucose and resistant starch content both exhibited an inverted U-shaped relationship with DSR (Fig. 1b and c). By contrast, fiber, tannin, and fat content had a U-shaped correlation with DSR (Fig. 1d to f). More notably, the turning point in all nonlinear curves occurred at the diet group with five plant species (all preferred by Brandt’s vole), and the gradual addition of further three nonpreferred plant species to the diet induced the reversal of the curve direction (Fig. 1d to f).

### Effects of DSR on postweaning performance.

**FIG 2:**
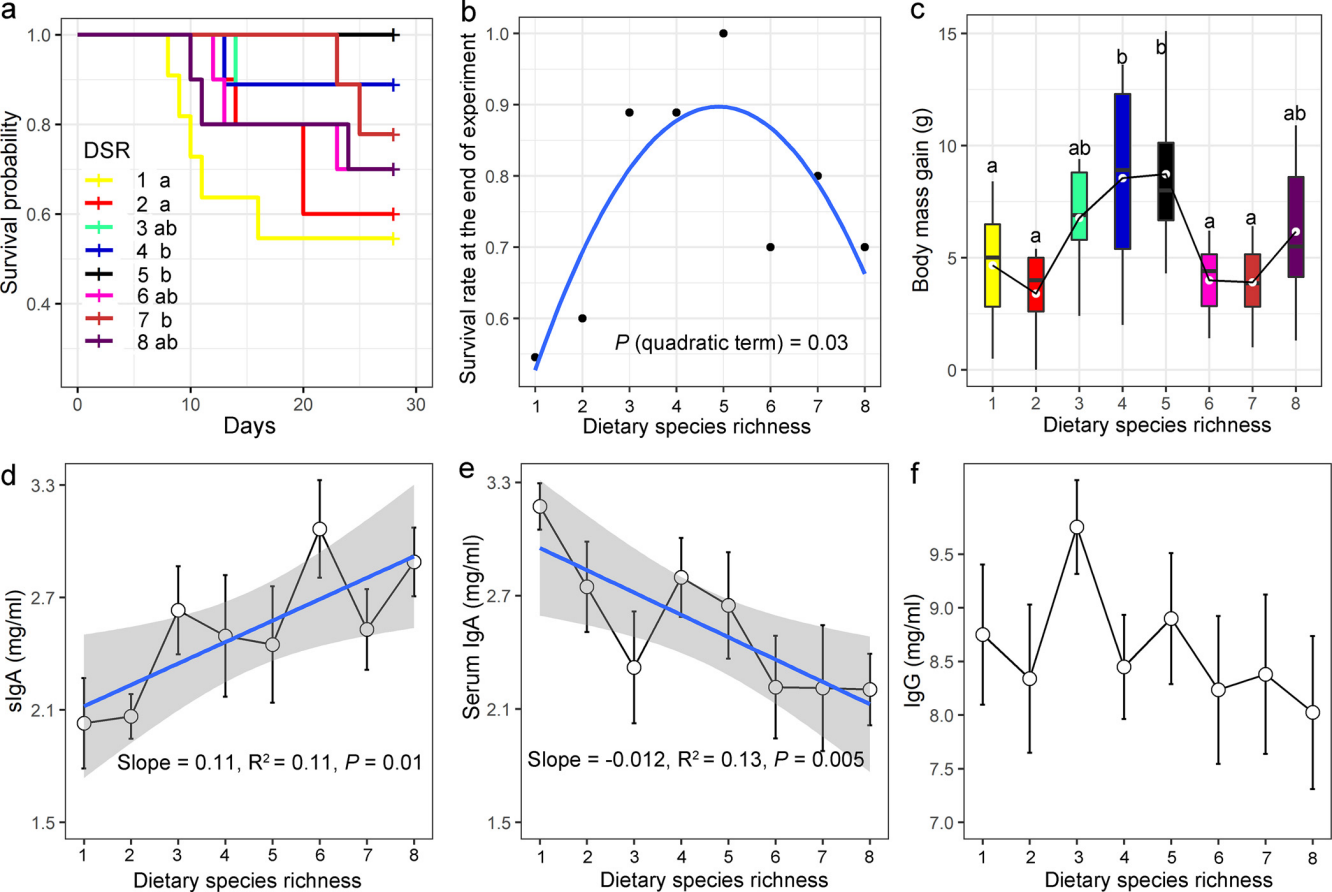
Effects of dietary species richness on the postweaning performance of Brandt’s vole. (a) Survival analysis of voles fed with diets of different plant species richness. (b) The inverted U-shaped relationship between survival rate of voles at the end of the experiment and the dietary species richness (DSR). (c) The inverted U-shaped relationship between body growth and the DSR shown by boxplot (displaying the minimum, first quartile, median, third quartile, and maximum for each group). Different letters denote significant difference between DSR treatments. (d) The content (mean ± se) of secretory immunoglobulin A (sIgA), (e) serum immunoglobulin A (IgA) and (f) serum IgG of voles in different DSR treatments.

Voles in low DSR treatments (DSR1 and DSR2) had significantly shorter overall survival time compared with those in some of high DSR treatments (DSR4, DSR5 and DSR7; Fig. 2a). The analysis of survival rate at the end of the experiment revealed an inverted U-shaped correlation with DSR (*P < *0.05; Fig. 2b; for group sizes, see Table S2 in the supplemental material). For the first five plant species, which are palatable food items highly preferred by Brandt’s vole, increasing the number of dietary plant species boosted vole survival rates. In contrast, for the last three plant species (i.e., nonpreferred species), the addition of further species into the diet largely reduced the survival rates (Fig. 2b). In a similar manner, an inverted U-shaped relationship was indicated between body weight gain and DSR (Fig. 2c). The highest values of both survival rate and body weight gain were at the level of 5 plant species for all treatments, which proves the importance of preferred food items. Furthermore, a strong positive relationship was demonstrated between secretory immunoglobulin A (sIgA) content and DSR (Fig. 2d). However, the serum immunoglobulin A (IgA) content was negatively associated with DSR (Fig. 2e). No significant correlations were detected between serum immunoglobulin G (IgG) and DSR (*P > *0.05, Fig. 2f). There was no significant difference in food intake between different dietary treatments (*P > *0.05, Table S2).

10.1128/mSystems.00979-21.5 TABLE S2 Group size of each dietary treatment before or after our experiment and average food intake for each different diet groups during the experiment. Download Table S2, DOCX file, 0.01 MB. Copyright © 2021 Li et al. 2021 Li et al. https://creativecommons.org/licenses/by/4.0/ This content is distributed under the terms of the Creative Commons Attribution 4.0 International license.

### Gut microbial community composition and assembly are shaped by DSR.

**FIG 3:**
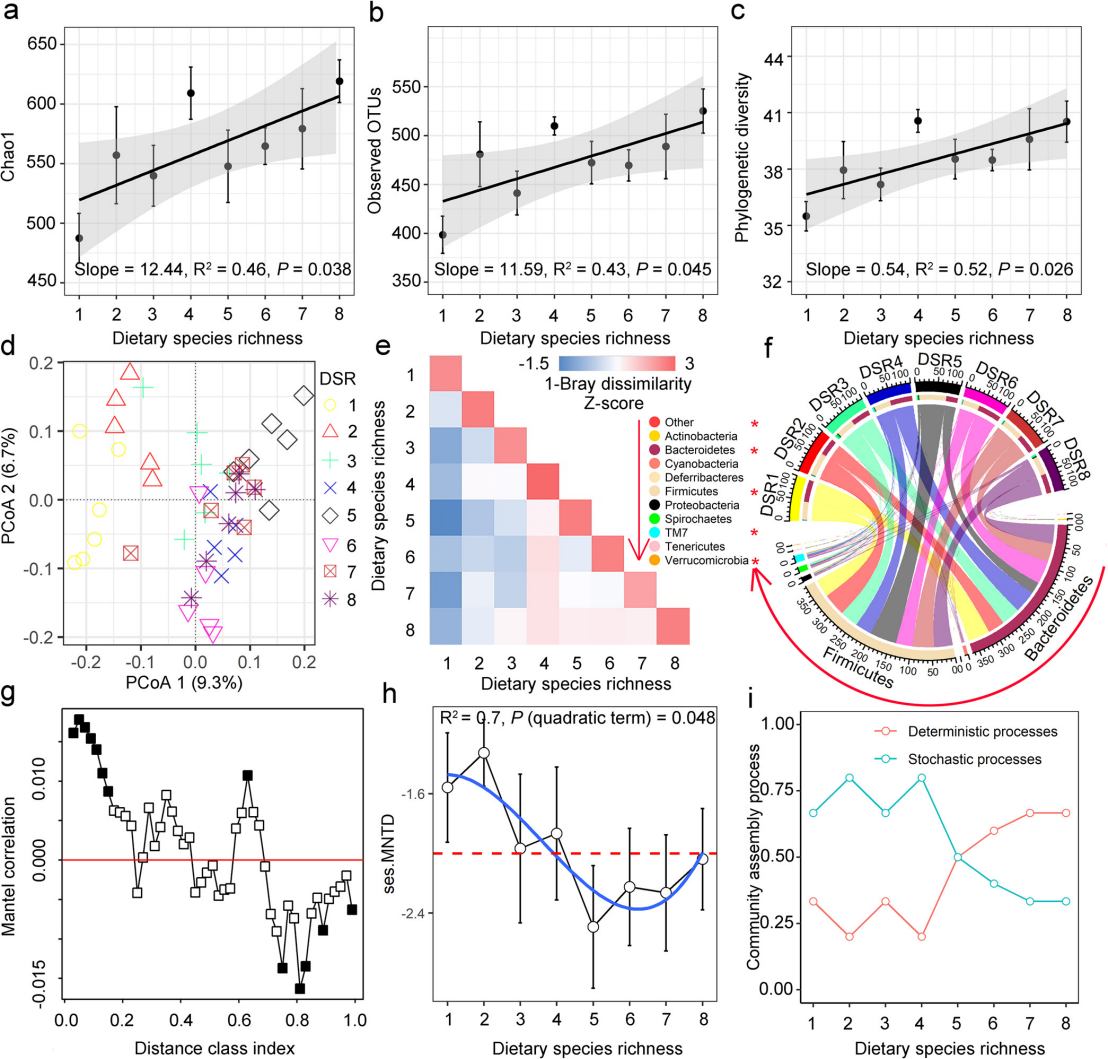
Effects of dietary species richness on the composition and assembly of gut microbiota of Brandt’s vole. (a–c) The positive relationship between dietary species richness and alpha diversity (i.e., Chao1, the number of observed operational taxonomic units [OTUs] and the phylogenetic diversity index, respectively) of gut microbiota. (d) Principal coordinates analysis (PCoA) of Bray-Curtis dissimilarity between samples colored by different DSR treatments. (e) The heatmap showing mean pairwise z-scores for similarity (i.e., 1 − Bray-Curtis dissimilarity) between dietary species richness (DSR) treatments. (f) Chord plot illustrating the gut microbial composition at the phylum level in different DSR treatments. Phyla are arranged in the direction of the arrows. (g) Mantel correlation between the Euclidean distance matrix of OTU niche values and phylogenetic distance matrix. Solid symbols denote significant (*P < *0.05, 999 permutations) correlations of phylogenetic signal, and open symbols denote nonsignificant correlations. (h) The u-shaped relationship between dietary species richness and the mean nearest taxon distance (SES.MNTD). (i) The relative contributions of deterministic processes and stochastic processes in microbial assembly of voles in different dietary species richness treatments.

The alpha diversity measures of gut microbiota (i.e., Chao1, observed operational taxonomic units [OTUs] and phylogenetic diversity) in Brandt’s voles increased with DSR (Fig. 3a to c). According to the Bray-Curtis distances, the principal coordinates analysis of taxonomic compositions of fecal samples showed a clear separation by diet based on different plant species richness (PERMANOVA, F = 2.05, R^2^ = 0.28, *P < *0.001; Fig. 3d). The gut microbial communities were observed to change with the shift from DSR3 to DSR4 treatments along with the increase of DSR but was relatively stable thereafter (Fig. 3e). The gut microbiota of Brandt’s voles was mainly composed of strains of two dominant bacterial phyla, Firmicutes and Bacteroidetes, which represented more than 93.5% of the total microbiome community. Further subdominant phyla included Proteobacteria, TM7, Cyanobacteria, Actinobacteria and Tenericutes (Fig. 3f). The ratio of Firmicutes/Bacteroidetes in vole microbiota peaked at the 5 plant species level treatment (DSR5, Fig. S1).

10.1128/mSystems.00979-21.2 FIG S1 Effect of dietary species richness (DSR) on the content of serum immunoglobulin G (IgG) (a), serum leptin (b), and the Firmicutes/Bacteroidetes ratio (c) of gut microbiota of Brandt’s voles. Download FIG S1, TIF file, 1.1 MB. Copyright © 2021 Li et al. 2021 Li et al. https://creativecommons.org/licenses/by/4.0/ This content is distributed under the terms of the Creative Commons Attribution 4.0 International license.

Significant phylogenetic signals (Mantel correlograms, *P < *0.05, solid circles) were found across relatively short phylogenetic distances (Fig. 3g), which enabled the use of phylogenetic turnover among the closest relatives to infer ecological processes. The values of SES.MNTD (i.e., Standardized effect size of mean nearest taxon distances) for gut microbial communities exhibited a U-shaped correlation with DSR, with its lowest mean value occurring at the DSR5 treatment group (Fig. 3h). The quantification of relative contributions of the deterministic and the stochastic processes in each DSR treatment group indicated that the community turnover of gut microbiota was principally governed by the stochastic processes for DSR1, DSR2, DSR3 and DSR4 groups. After the addition of nonpreferred plants to the diet, however, the deterministic processes mainly shaped the assembly of vole gut microbiota (i.e., DSR6, DSR7 and DSR8 treatment groups; Fig. 3i).

### Structure and function of gut microbiome and their associations with nutrient metabolism and immunity.

**FIG 4:**
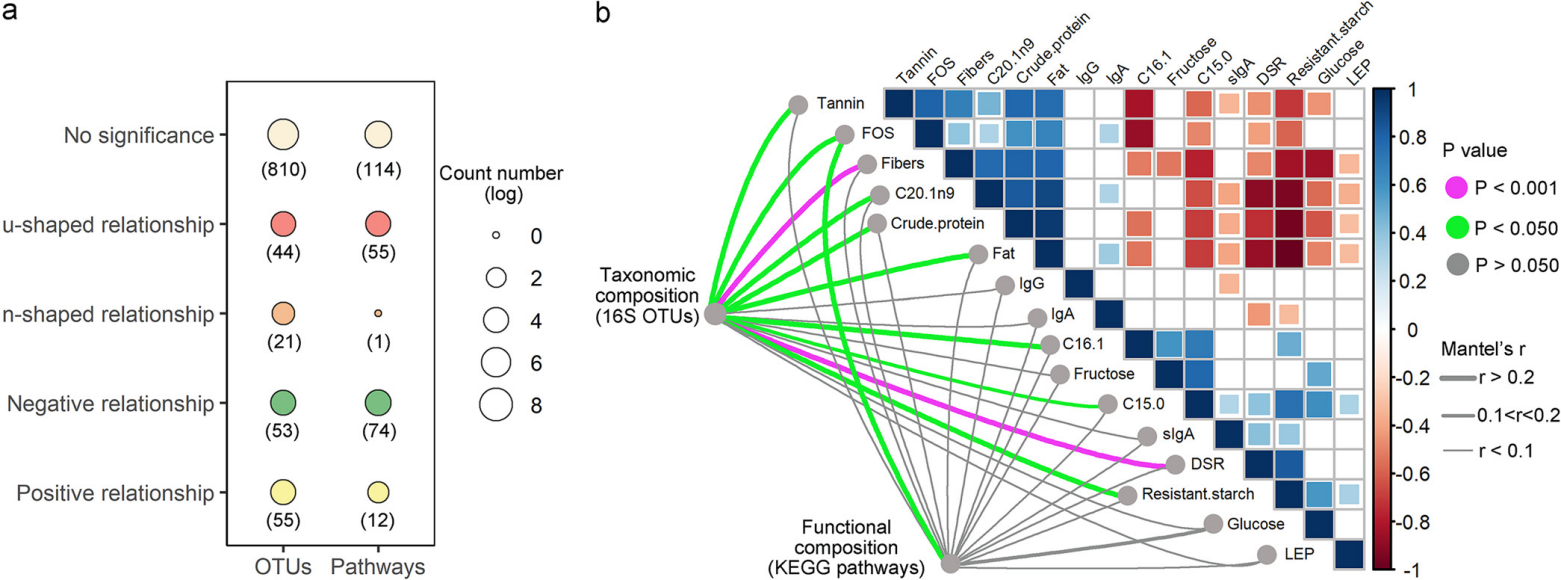
Feedback mechanisms of gut microbial communities to dietary species richness. (a) Summary of the relationships between dietary species richness and taxonomic composition (i.e., OTUs), and dietary species richness and functional composition (i.e., metabolic pathways). The number in the bracket indicates the number of OTUs or metabolic pathways. (b) Pairwise comparisons of nutrient and immune aspects with a color gradient denoting Pearson’s correlation coefficient. Taxonomic and functional community structures were related to each environmental factor by Mantel tests. Edge width denotes the Mantel’s r value for the corresponding distance correlations, and edge color indicates the statistical significance.

Regression analysis was used to measure the relationship between OTUs and microbial function pathways in different DSR treatments. Specifically, for 173 of the 983 OTUs, a diverse response pattern to DSR was established (for more details, see Table S3 and S4 in the supplemental material), with 44 exhibiting a U-shaped relationship, 21 indicating an inverted U-shaped relationship, 53 presenting a negative relationship and 55 featuring a positive relationship (Fig. 4a). Among the total of 256 functional pathways, 142 functional pathways identified were differentially related to DSR (for more details, see Table S5 & S6), with 55 pathways exhibiting a U-shaped relationship, 1 pathway having an inverted U-shaped relationship, 74 pathways presenting a negative relationship (e.g., lysine biosynthesis I and tryptophan biosynthesis) and 12 pathways showing a positive relationship (e.g., methionine biosynthesis I and III) with DSR (Fig. 4a). Both for OTUs and functional pathways, the number of linear responses far outweighed the number of nonlinear responses.

10.1128/mSystems.00979-21.6 TABLE S3 OTUs that have significantly linear relationship with dietary species richness (DSR). Download Table S3, XLSX file, 0.02 MB. Copyright © 2021 Li et al. 2021 Li et al. https://creativecommons.org/licenses/by/4.0/ This content is distributed under the terms of the Creative Commons Attribution 4.0 International license.

10.1128/mSystems.00979-21.7 TABLE S4 OTUs that have significantly nonlinear relationship with dietary species richness (DSR). Download Table S4, XLSX file, 0.02 MB. Copyright © 2021 Li et al. 2021 Li et al. https://creativecommons.org/licenses/by/4.0/ This content is distributed under the terms of the Creative Commons Attribution 4.0 International license.

10.1128/mSystems.00979-21.8 TABLE S5 The functional pathways that have significant linear relationship with dietary species richness (DSR). Download Table S5, XLSX file, 0.02 MB. Copyright © 2021 Li et al. 2021 Li et al. https://creativecommons.org/licenses/by/4.0/ This content is distributed under the terms of the Creative Commons Attribution 4.0 International license.

10.1128/mSystems.00979-21.9 TABLE S6 The functional pathways that have significant nonlinear relationship with dietary species richness (DSR). Download Table S6, XLSX file, 0.02 MB. Copyright © 2021 Li et al. 2021 Li et al. https://creativecommons.org/licenses/by/4.0/ This content is distributed under the terms of the Creative Commons Attribution 4.0 International license.

To identify the drivers of nutrition and immunology, the dissimilarities of microbial community taxonomic and functional structure were correlated with those of nutrients and immune measurements. Any structural shifts were closely linked to external factors, including levels of tannin, fructo-oligosaccharides (FOS), fiber, crude protein, fat and resistant starch, as revealed by the Mantel test (Fig. 4b). Overall, the FOS content had the strongest correlation with both the taxonomic and functional composition of Brandt’s vole microbiome (Fig. 4b). In addition, DSR was a primary driver of taxonomic structure of the microbial community, although no significant correlation was established between DSR and the functional structure of the microbial community.

### Fecal amino acid and short chain fatty acid levels are influenced by DSR.

**FIG 5:**
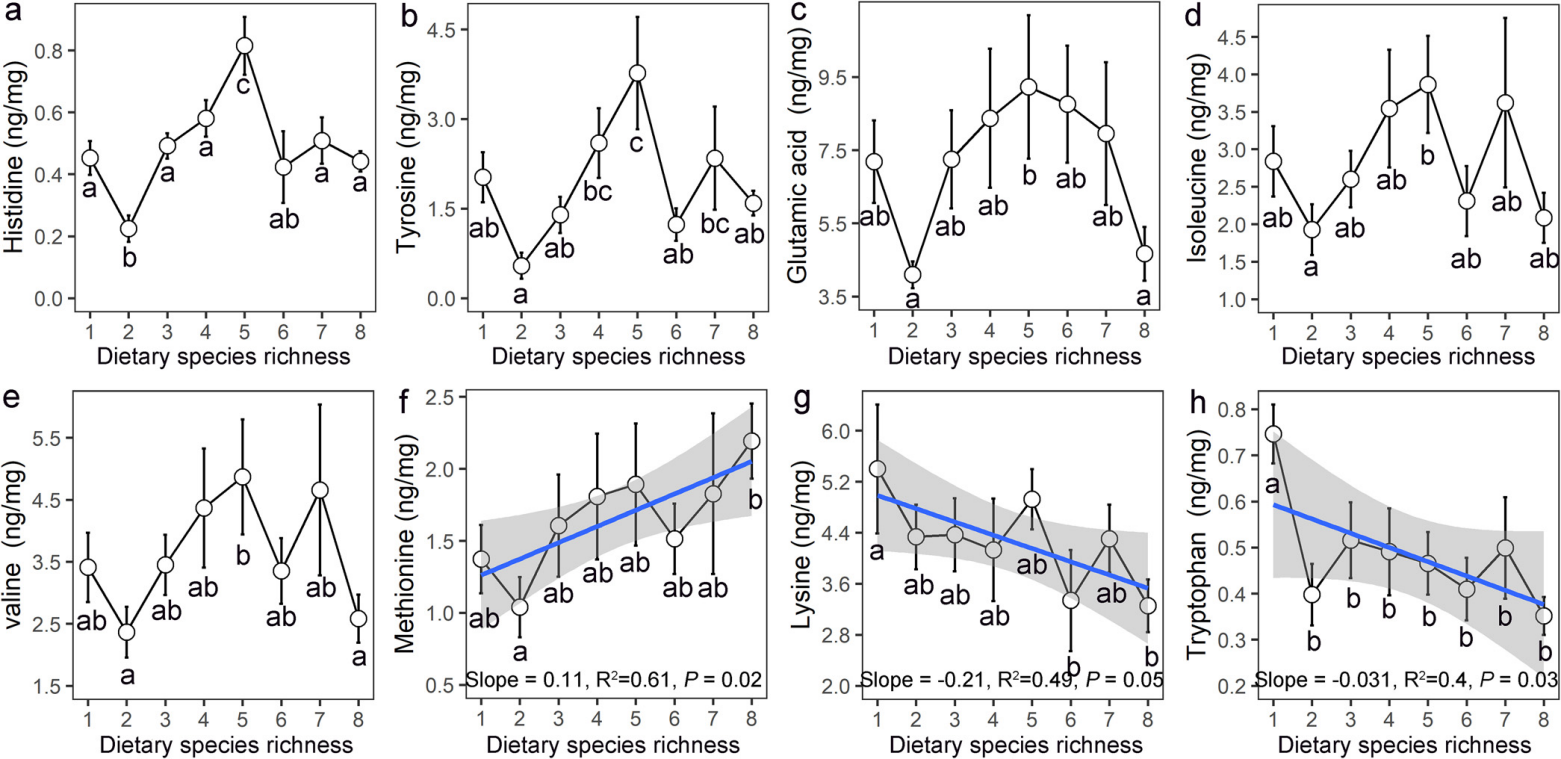
Effect of dietary species richness (DSR) on the concentration of fecal amino acids (mean ± SE). Different letters denote significant difference between DSR treatments. Significant correlations between DSR and fecal amino acids are shown by adding a trending line.

**FIG 6:**
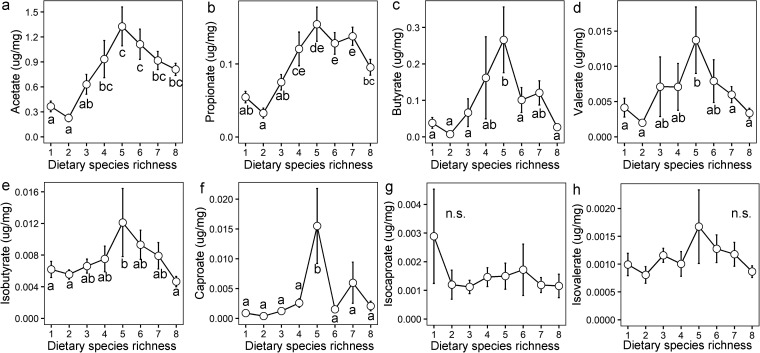
Effect of dietary species richness on the concentration of fecal short-chain fatty acids (mean ± SE). Different letters denote significant difference between dietary species richness (DSR) treatments. The n.s. means not statistically significant at *P < *0.05 between DSR treatments.

To validate the DSR-induced changes in the metabolic functions of gut microbiome community, the levels of all 20 amino acids and 8 short chain fatty acids of fecal samples from various DSR treatments were quantified. Eight of 20 amino acids were found to significantly correlate with DSR, including 5 amino acids (histidine, tyrosine, valine, glutamic acid and isoleucine) featuring an inverted U-shaped relationship with DSR; all of the corresponding peak points were associated with the DSR5 treatment (Fig. 5a to e). Dietary species richness had a negative relationship with lysine and tryptophan and a positive relationship with methionine (Fig. 5f to h), which were consistent with the shift of metabolic functions in the microbial community. The short-chain fatty acids (SCFAs) of fecal samples mostly consisted of acetate (79.8%), propionate (10.7%) and butyrate (7.2%), and other subdominant types including isobutyrate, isovalerate, valerate, isocaproate and caproate (Fig. 6). These eight SCFAs (except for isocaproate and caproate) all presented an inverted U-shaped relationship with DSR, with all peak points at the DSR5 treatment (Fig. 6a to h).

## DISCUSSION

Gut microbial communities are readily influenced by host diets and can exert strong positive or negative effects on host nutrition and body development (4), as well as contribute to the modulation of animal behavior (35) and immune system maturation (36). Therefore, understanding the links between gut microbial community assembly and maintenance of its diversity is an important tool for explaining variations in the individual performance and population size of the host. In this paper, a detailed characterization of correlations between gut microbial diversity (GMD), microbial community assembly and DSR is provided, as well as that of the mechanisms of how such correlations influence the performance of the animal host. As such, our study is one of the few research to date that have focused on the DSR-GMD relationship in a wild vertebrate animal species.

### Host performance response to DSR.

It is now widely accepted that, due to the nutrient complementation (37) and toxin dilution (38) by dietary mixing, high DSR has positive linear effects on generalist herbivore performance, while a single-species diet can easily lead to poor individual performance, which translates to a sharp decline in small herbivore populations (39). However, in this study, the performance of Brandt’s vole, measured as survival time/rate and body growth, varied nonlinearly between treatments with different DSR diets. Consistently with previous studies, Brandt’s voles exhibited better performance when offered a multiple-species diet compared with being restricted to only one or two plant food items, which can be explained by the enhanced nutrient balance and adequacy in mixed diets. In contrast to previous studies, however, the direction of effects of DSR (either positive or negative) depended on the percentage of preferred plant species in our study. Specifically, a positive linear effect of DSR was present when the food items were all preferred plant species, while the gradual addition of nonpreferred food items into diets exerted a negative effect. Herbivores usually show preferences for plants species that are nutritious or have low levels of toxins. Thus, low preference-ranked foods reflect low rates of nutritional value. In our study, the addition of nonpreferred plant species into diets resulted in elevated tannin content (thereby inhibiting plant protein digestion), but decreased glucose content (serving as an energy reserve) and resistant starch (serving as prebiotics).

Nonmonotonic interactions are largely responsible for the complexity of ecological processes, with transitions among positive, negative, and neutral effects that have received much attention by theoretical and field ecologists (40–42). The nonlinear effect of DSR on the performance components of Brandt’s vole may be due to the nonadditive interactions of diet items consumed in a mixture. On the one hand, one diet item may negatively impact the intake and digestion of a different diet item. For example, a plant species with high tannin content can decrease the digestibility of protein in other plant species with low tannin content. On the other hand, the combination of nutrients in two plants might be beneficial in that the absorption capacity of both will be improved. For example, our previous study demonstrated that high fructose content in diets can positively affect the body growth of Brandt’s vole (4), while its absorption capacity was relatively low and could be enhanced by the addition of glucose, especially when fructose to glucose ratio was 1:1 (43). With an increasing proportion of preferred plant species in the diet, this ratio showed parallel increase and gradually approached the optimal value, while once nonpreferred plant species were added to the diet, it tended to deviate from its optimum (Fig. S2). Consequently, preference-based DSR diets may induce differences in nutrient digestion and absorption, and eventually contribute to the alteration of performance components in a nonlinear fashion.

10.1128/mSystems.00979-21.3 FIG S2 The ratio of fructose to glucose in different diets with different dietary species richness (DSR). Fructose can be best absorbed by small intestinal when it is taken with equal glucose (the red line). Download FIG S2, TIF file, 0.04 MB. Copyright © 2021 Li et al. 2021 Li et al. https://creativecommons.org/licenses/by/4.0/ This content is distributed under the terms of the Creative Commons Attribution 4.0 International license.

In our study referred to above, the curve of relationship between microbiota-generated metabolites (i.e., SCFAs including acetate, propionate, butyrate, and valerate) and DSR exhibited an inverted U-shape. A growing number of evidence indicate that SCFAs can act as energy substrates regulating key metabolic pathways, with the capacity to substantially improve the survival and growth of animals and humans (4, 44). This nonlinear response of SCFAs to DSR may explain the similar response type of Brandt’s vole survival and body growth to DSR. In addition, the inverted U-shaped relationship between performance components and DSR may also be the result of the joint effect of linear relationships (both with positive and negative associations) between physiological or metabolic traits and DSR (42). In this study, diets with increasing DSR elevated the methionine content while reducing the lysine content. Given that these are both key amino acids in facilitating the body growth of animals (45), the joint effect may finally result in the nonlinear response of body growth to DSR. In a similar fashion, many other linear relationships (both positive and negative) between gut microbiota composition or functions and DSR were established in our study (Fig. 4a), which were involved in various physiological and metabolic processes, and may also have caused the nonlinear response of performance components of Brandt’s vole.

sIgA has an essential function in the defense against pathogenic microorganisms in the gut, maintaining immune tolerance to nonpathogenic intestinal bacteria and establishing intestinal homeostasis (36). It has been well-established that sIgA and gut microbiota diversity have a strong positive relationship, although the direction of causality identified by different studies shows variation. On the one hand, exposure to a high diversity of gut microbiota antigens and derived metabolites (e.g., SCFAs) in the mucus can train and regulate the development and maturation of the mucosal sIgA system in early life; an example is early colonization with highly diverse *Bifidobacterium* microflora causing a buildup of sIgA content (46). However, sIgA abundance and diversification can also regulate gut microbiota composition and diversity by mediating the entrance of noninvasive bacteria into Peyer’s patches (47). In our study, voles receiving higher DSR diets and thus harboring a larger variety of harmful microorganisms also exhibited raised levels of intestinal sIgA response, although the underlying mechanism is unclear. Furthermore, diets with increased DSR altered the host systemic humoral immunity in a manner that reduced serum IgA levels. This inverse relationship between serum IgA and sIgA may be due to the tradeoff between mucosal and systemic immune response. If the sIgA system fails as the first line of defense allowing antigens to pass through the epithelium, serum IgA is subsequently activated to remove the pathogens from the circulatory system and body tissues (48, 49).

### Effects of DSR on gut microbial diversity and community assembly.

Higher gut microbial species diversity is generally linked to more functional redundancy and better health (29). A more diverse diet may create a more varied nutritional habitat in the gut favoring a wider range of microbial taxa (50). Our results showed that the alpha diversity of gut microbial community of Brandt’s voles consistently increased with a higher DSR diet. According to the phylogenetic analysis, stochastic processes dominated microbial community assemblages in the vole gut ecosystem in a low DSR diet. However, after the addition of nonpreferred plant species into the vole diet, deterministic processes tended to intensify. This pattern is assumed to be largely the result of changes in sIgA and prebiotics content (e.g., resistant starch and fructo-oligosaccharides or FOS) with the increase of DSR of diets. The sIgA and prebiotics may play essential roles in determining which bacteria colonize the gut. For example, sIgA antibodies can bind to Salmonella *typhimurium* and inhibit Salmonella invasion (47). Moreover, resistant starch supplementation can promote the growth of Bifidobacterium adolescentis and Ruminococcus bromii and thus increase butyrate production (51). High FOS consumption can elevate the relative abundance of probiotic microbes (e.g., Bifidobacterium and Lactobacillus) and butyrate-producing microbes such as *Oscillospira*, Ruminococcus and Faecalibacterium (52).

### Implications for population dynamics.

Due to the importance of small rodent species in maintaining biodiversity and ecosystem services, their population dynamics have fascinated ecologists all over the world for nearly 1 century. Meanwhile, the mechanisms of abrupt population collapse after outbreaks are not yet fully understood (53). The intense herbivory by rodent during population peak phase can create a top-down control of plant community composition and species richness, likely resulting in a decline of preferred plants for that species. Our results imply that a reduction in the ratio of preferred food items in the diet may exert negative effects on newborn vole performance though the regulation of community assemblage and metabolic functions of the gut microbiome. This may account for the low phase in the population cycle, although future field trials are needed to support this hypothesis.

In summary, our results represent an example of gut microbial assembly driven by the interaction between dietary species richness and diet preference. This study, which is believed to be among the first few manipulative trials aimed at clarifying these interactions, revealed a nonlinear performance consequence of such interactions for a host animal. It is concluded that the performance of generalist herbivores may diminish in plant communities featured by increased total plant species richness but also by a scarcity of preferred food items, which, through trophic transfer effects, may pose a threat to ecosystem stability. Given the fast-growing interest in therapeutic diets to treat dysbiosis and to improve health conditions (50), our study highlights the need to consider not just the variety of consumed food but also the principles of rational nutrition.

## MATERIALS AND METHODS

### Study design and background.

The field trial was conducted at the Inner Mongolia Research Station of Animal Ecology (44°11′ N, 116°27′ E) in Xilinhot, Inner Mongolia, China. The area belongs to a typical steppe habitat of Inner Mongolia, which is dominated by the perennial grasses L. chinensis and S. krylovii (both are C_3_ species). Other subdominant plant species include the C_3_ species Allium polyrhizum, Medicago sativa, Phlomis dentosa, and Convolvulus ammannii, as well as the C_4_ species C. squarrosa and C. aristatum. Ongoing climate warming is already causing functional group composition shifts by favoring the success of C_4_ over C_3_ species in the Inner Mongolia grassland (13). The primary source of human disturbance in the region is livestock grazing, which affects plant species composition, plant species diversity (54) and small mammal populations (14). Nonetheless, moderate grazing has been reported to induce the enhancement of species diversity by improving light and space availability, and by suppressing the growth of dominant plant species (55).

### Diet manipulation experiment.

Owing to the joint effects of climate warming and moderate livestock grazing, the DSR increased while the ratio of preferred plants in the diet of Brandt’s vole decreased in the study area. To simulate the shift in the composition and diversity of food items and assess these effects on the performance of the species in question, a diet manipulation experiment was performed with eight native plant species involved. These are all common food species of Brandt’s vole in the typical Inner Mongolia steppe and are ranked in descending order of preference as follows (56, 57): L. chinensis *>* M. sativa *>* A. polyrhizum *>* C. ammannii *>* S. krylovii *>* C. squarrosa *>* C. aristatum *>* P. dentosa. From the vole’s dietary perspective, the first five are the most preferred species, while the last three are nonpreferred species. The DSR gradient was divided into eight levels (Table S1 in the supplemental material), corresponding to DSR1, DSR2, DSR3, DSR4, DSR5, DSR6, DSR7, and DSR8 treatment, respectively. All treatment combinations of plant species were created based on the major sources of food preferred by or available to Brandt’s vole. The major components of Brandt’s vole’s diet (4), L. chinensis and S. krylovii, were treated as the base dietary items. The gradient of DSR was created by a gradual addition of plant species with lower preference to the base diet.

10.1128/mSystems.00979-21.4 TABLE S1 Plant species offered to Brandt's voles for each dietary species richness (DSR) treatment. The check mark symbol indicates that the plant species is included in the corresponding DSR treatment. Download Table S1, DOCX file, 0.02 MB. Copyright © 2021 Li et al. 2021 Li et al. https://creativecommons.org/licenses/by/4.0/ This content is distributed under the terms of the Creative Commons Attribution 4.0 International license.

Fresh plants were collected in the field near the research station and were oven dried at 40°C for 3 days. The dry samples were grounded into uniformly small particles and mixed in equal proportions of the selected plants of each DSR treatment to prepare food sticks for the voles. In late July of 2018, when newborn voles had just weaned, they were live-trapped in the grassland around Erhebaolige town. These individuals were individually housed in 25.5 × 15 × 13.5 cm polypropylene cages at the research station under natural photoperiodic conditions and were provided with corncob bedding material and water and rabbit chow *ad libitum* (Beijing Huafukang Biotechnology Co. Ltd., 14% protein, 15% crude fiber, 3% fat). This diet was maintained for 1 week prior to the start of the experiment as a 1-week acclimation period to ensure that all voles were healthy (accordingly, no death or weight loss were observed during this period), and to ensure that all animals exhibited similar gut microbiota profiles, thus reducing natural variation within the live-captured population. A total of 80 newly-weaned voles (22 ± 0.8 g) were randomly assigned to different DSR treatments. They were raised individually with food in abundance (i.e., of the respective special diet) and water for 4 weeks. Their survival status was checked and recorded daily for each individual. At Week 3, voles received a single subcutaneous injection of keyhole limpet haemocyanin (KLH, Sigma 7017; 0.2 mg in 0.2 ml sterile saline) to assess their humoral immune response to DSR. At the end of the experiment, fresh feces were collected and frozen at –80°C for future DNA extraction, sequencing and metabolism analysis. Voles were eventually anesthetized with sodium pentobarbital (1 mg/10 g body mass) and immediately weighed. Blood samples were obtained by decapitation and centrifuged at 4°C for 30 min at 4000 rpm. Blood serum samples were stored at –20°C until anti-KLH IgA assay and IgG concentration measurement. The concentrations of nutritional indexes (including fibers, crude protein, fructose, glucose, resistant starch and fructo-oligosaccharides) and secondary metabolic compounds (i.e., tannin and silicon) of each plant species were determined to assess their association with gut microbiome and vole performance for different DSR treatments. The taxonomic composition and functional profile of microbial communities of voles in different DSR treatments were characterized using 16S and shotgun metagenomics. To validate the significant changes in specific metabolic functions, the targeted metabolome analysis (amino acids and short chain fatty acids) of fecal samples was performed between DSR treatments. The experimental process for the measurement of the above-mentioned indexes strictly referred to methods utilized in our previous studies (4, 34). The experiments were approved by the Institutional Animal Care and Use Committee of the Institute of Zoology, Chinese Academy of Sciences (IOZ-IACUC-2020-074). All experiments were performed in accordance with relevant guidelines and regulations.

### DNA extraction, sequencing and read processing.

We extracted the total DNA from fresh fecal samples by using Tiangen DNA kit (Tiangen Biotech, Beijing, China) according to the manufacturer's instructions. DNA concentrations was assessed by using a Qubit 2.0 Fluorometer (Invitrogen, Life Technologies). PCR amplification of the V3–V4 hypervariable region of the bacterial 16S rRNA gene was performed to investigate the profiles of the microbial composition by using the universal primers PCR 341F (5′-CCTAYGGGRBGCASCAG-3′) and 806R (5′-GGACTACNNGGGTATCTAAT-3′). PCRs were carried out with the following cycling conditions (98°C, 60 s; 30 × [98°C, 10 s; 50°C, 30 s; 72°C, 30 s]; 72°C, 5 min) and checked on a 2% agarose gel. Sequencing libraries were prepared using TruSeq DNA PCR-Free Sample Preparation Kit (Illumina, USA) and NEBNext Ultra™ DNA Library Prep Kit for Illumina (NEB, USA) for 16S amplicon sequencing and shotgun metagenomic sequencing, respectively, according to the manufacturer's instructions and unique dual indexing adaptors were added to attribute sequences to each sample. 16S amplicon sequencing was performed on an Illumina Miseq Platform (2 × 300-bp base-paired reads). Shotgun metagenomic sequencing of the fecal DNA was performed to profile the microbial metabolic diversity on an Illumina NovaSeq 6000 sequencing platform, resulting at least 7 Gb of 150 bp paired-end reads per sample by using standard Illumina sequencing protocols.

### Bioinformatic analysis.

The analysis of raw sequencing reads was performed by using the Quantitative Insight Into Microbial Ecology (QIIME, version 1.9.1) pipeline (58). We merged raw reads by FLASH software (version 1.2.7) and eliminated all singleton and chimeric sequences by using USEARCH11 (59) based on the UCHIME algorithm. The remaining reads were split into OTUs at threshold 97% and then aligned against the Greengenes database to remove nonbacterial reads. Taxonomic assignment was performed using Greengenes (gg13.8) reference databases and the phylogenetic tree was constructed by the make_phylogeny.py script in QIIME for further phylogenetic analysis. Alpha-diversities (number of OTUs, Chao 1 index, Shannon index and Faith’s phylogenetic diversity) and beta-diversity (Bray-Curtis distances between samples) were calculated for the rarefied OTU table by using the alpha_diversity.py script and the beta_diversity.py script in QIIME respectively.

For the metagenomic analysis, quality control was performed by using the KneadData pipeline (https://github.com/biobakery/kneaddata). MetaPhlAn2 was used to generate information about taxonomic composition. The abundance of gene pathways for metagenomic reads were characterized using the HUMAnN2 pipeline (a pipeline for efficiently and accurately profiling the metabolic potential of a microbial community) (60) with the DIAMOND (a fast and sensitive protein aligner) (61).

### Phylogenetic analysis.

We used the β mean nearest taxon distance (βMNTD) measure and nearest taxon index (βNTI) to explore mechanisms underlying community assembly of microbiota in the voles with different diets in “picante” R package (62, 63). βMNTD indicates nearest taxon distance between all pairs of OTUs and βNTI measures the deviation of observed βMNTD from mean expected βMNTD in a null model. According to Stegen et al. (2013), βNTI > 2 or βNTI <-2 means co-occurring OTUs are more closely or distantly related than expected by chance, indicating the dominance of deterministic processes (phylogenetic clustering or phylogenetic overdispersion). By contrast, if βNTI values are between -2 and 2, then the microbial community is predominated by stochastic processes. To enable the use of phylogenetic information to infer underlying ecological processes, significant phylogenetic signal across relatively short distances is required (i.e., phylogenetic distances between taxa approximate their environmental niche differences) (64). First, the environmental-optimum for each OTU in the gut was calculated with respect to dietary nutrition as in Stegen et al. (2012) (63). Then, between-OTU nutrition optima differences were calculated as Euclidean distances using optima for all the nutrition variables. Lastly, we performed a Mantel correlogram to evaluate the correlation coefficients using the Vegan package in R (65).

### Statistical analysis.

All statistical analyses were conducted using R version 4.0.3 (66). A Cox proportional hazards model was fit in R to examine the effect of DSR on the survival time of voles with the coxph function from the Survival package. We used polynomial regressions to examine the bitonic relationship between the relative abundance of OTUs and DSR, and the relative abundance of functional pathways and DSR. The *P* values were adjusted using the false discovery rate (FDR) correction for multiple hypothesis testing with p.adjust function from the Stats package (67). ANOVA was run through the aov function from the Stats package to compare the differences in various measurements (i.e., body growth, nutrients, immunity indices, and small metabolites) between DSR treatments. We performed a PERMANOVA test based on the Bray-Curtis dissimilarity metric with the adonis function in the R package Vegan.

### Availability of data.

All 16S sequence data and metagenome sequence data used in this study are available at the NCBI Sequence Read Archive (https://www.ncbi.nlm.nih.gov/) under BioProject ID PRJNA722573 and PRJNA723632, respectively.
