# Supplementary material for: The Role Transition of Dietary Species Richness in Modulating the Gut Microbial Assembly and Postweaning Performance of a Generalist Herbivore
Source: mSystems. 2021 Nov 2;6(6):e00979-21. doi: 10.1128/mSystems.00979-21 (PMC8562480; doi:10.1128/mSystems.00979-21)
Supplement: TABLE S2 [file msystems.00979-21-st002.docx]

| **Table S2** | | | |
| --- | --- | --- | --- |
| Treatments | No. of voles at the beginning of experiment | No. of voles at the end of experiment | Average food intake (g/day) |
| DSR1 | 11 | 6 | 5.77 |
| DSR2 | 10 | 6 | 5.93 |
| DSR3 | 9 | 8 | 5.87 |
| DSR4 | 9 | 8 | 5.95 |
| DSR5 | 10 | 10 | 5.93 |
| DSR6 | 10 | 7 | 5.86 |
| DSR7 | 10 | 8 | 5.41 |
| DSR8 | 10 | 7 | 5.47 |
