# Supplementary figures and images for: The Role Transition of Dietary Species Richness in Modulating the Gut Microbial Assembly and Postweaning Performance of a Generalist Herbivore
Source: mSystems. 2021 Nov 2;6(6):e00979-21. doi: 10.1128/mSystems.00979-21 (PMC8562480; doi:10.1128/mSystems.00979-21)

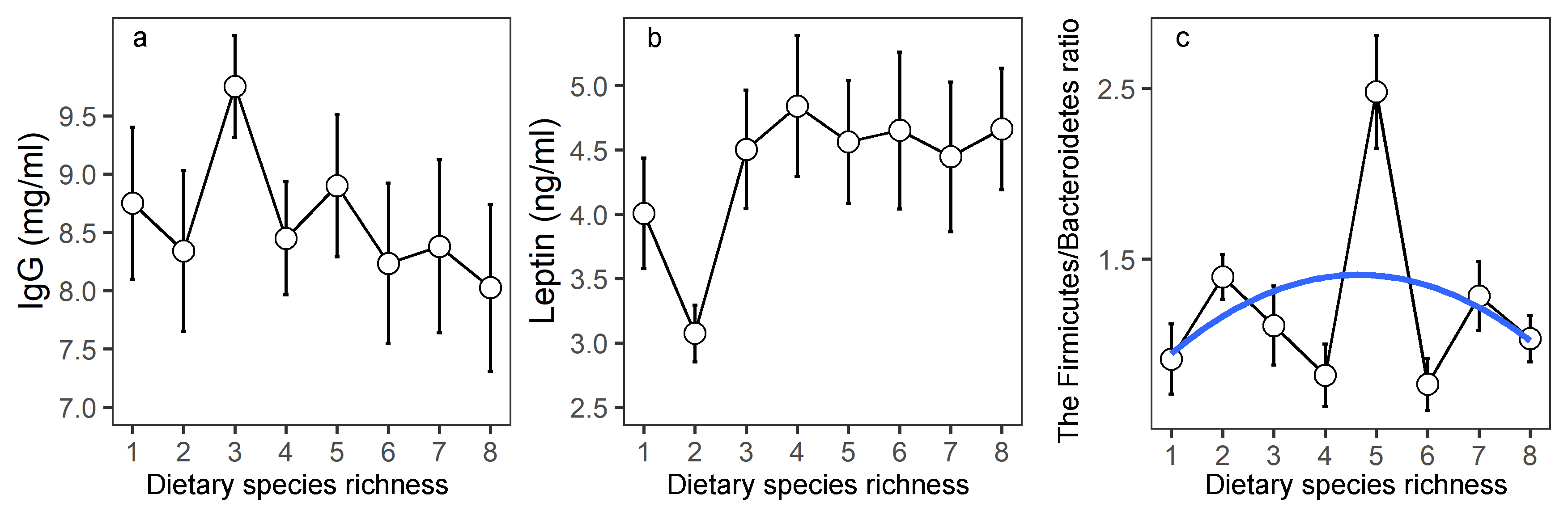

Supplement: FIG S1 [file msystems.00979-21-sf001.tif]

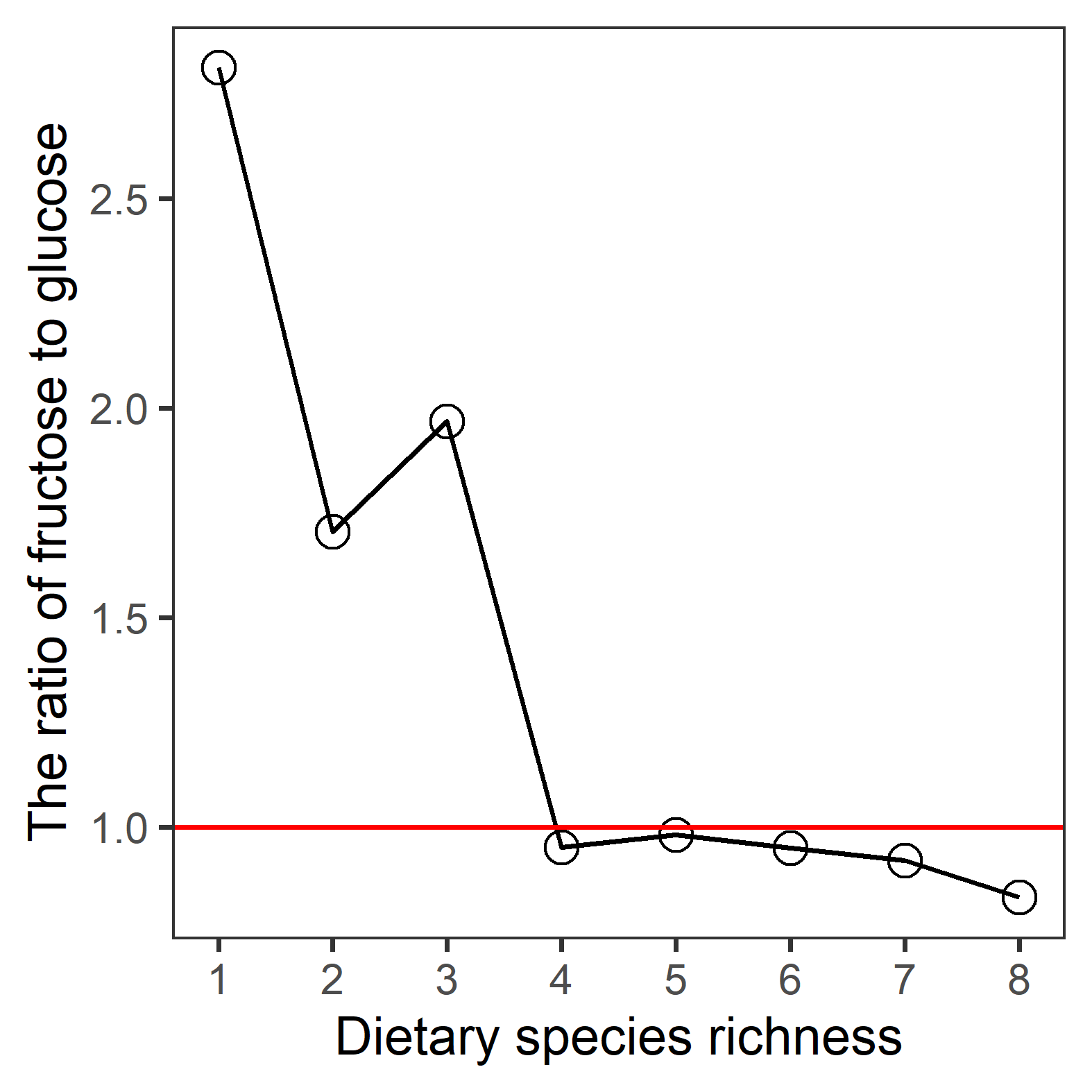

Supplement: FIG S2 [file msystems.00979-21-sf002.tif]
