# Supplementary material for: The Role Transition of Dietary Species Richness in Modulating the Gut Microbial Assembly and Postweaning Performance of a Generalist Herbivore
Source: mSystems. 2021 Nov 2;6(6):e00979-21. doi: 10.1128/mSystems.00979-21 (PMC8562480; doi:10.1128/mSystems.00979-21)
Supplement: TABLE S1 [file msystems.00979-21-st001.docx]

| **Table S1** | | | | | | | | | |
| --- | --- | --- | --- | --- | --- | --- | --- | --- | --- |
| DSR treatment | The preferred species | | | | |  | The non-preferred species | | |
|  | *L. chinensis* | *S. krylovii* | *M. sativa* | *A. polyrhizum* | *C. ammannii* |  | *C. squarrosa* | *C. aristatum* | *P. dentosa* |
| DSR1 | √ |  |  |  |  |  |  |  |  |
| DSR2 | √ | √ |  |  |  |  |  |  |  |
| DSR3 | √ | √ | √ |  |  |  |  |  |  |
| DSR4 | √ | √ | √ | √ |  |  |  |  |  |
| DSR5 | √ | √ | √ | √ | √ |  |  |  |  |
| DSR6 | √ | √ | √ | √ | √ |  | √ |  |  |
| DSR7 | √ | √ | √ | √ | √ |  | √ | √ |  |
| DSR8 | √ | √ | √ | √ | √ |  | √ | √ | √ |
